# Can they Feel? The Capacity for Pain and Pleasure in Patients with Cognitive Motor Dissociation

**DOI:** 10.1007/s12152-018-9361-z

**Published:** 2018-05-21

**Authors:** Mackenzie Graham

**Affiliations:** Uehiro Centre for Practical Ethics, Wellcome Centre for Ethics and Humanities, Oxford University, Oxford, UK

**Keywords:** Pain, Pleasure, Unresponsive wakefulness syndrome, Disorders of consciousness, Well-being, Sentience, Neuroimaging, Cognitive motor dissociation

## Abstract

Unresponsive wakefulness syndrome is a disorder of consciousness wherein a patient is awake, but completely non-responsive at the bedside. However, research has shown that a minority of these patients remain aware, and can demonstrate their awareness via functional neuroimaging; these patients are referred to as having ‘cognitive motor dissociation’ (CMD). Unfortunately, we have little insight into the subjective experiences of these patients, making it difficult to determine how best to promote their well-being. In this paper, I argue that the capacity to experience pain or pleasure (sentience) is a key component of well-being for these patients. While patients with unresponsive wakefulness syndrome are believed to be incapable of experiencing pain or pleasure, I argue that there is evidence to support the notion that CMD patients likely *can* experience pain and pleasure. I analyze current neuroscientific research into the mechanisms of pain experience in patients with disorders of consciousness, and provide an explanation for why CMD patients likely can experience physical pain. I then do the same for physical pleasure. I conclude that providing these patients with pleasurable experiences, and avoiding subjecting them to pain, are viable means of promoting their well-being.

## Introduction

‘Unresponsive wakefulness syndrome’ (UWS), commonly referred to as the ‘vegetative state’, is a disorder of consciousness caused by severe traumatic or anoxic brain injury [1]. (Throughout the paper, I will refer to patients diagnosed as vegetative as UWS/VS, to reflect the recent change in taxonomy). While patients in this state appear to go through periods of wakefulness and sleep, they show no evidence of being aware of themselves or their surroundings [2]. However, functional neuroimaging has been used to demonstrate that a proportion of patients diagnosed as UWS/VS can modulate their brain activity in response to command, thus demonstrating that they are, in fact, aware [3–6]. The presence of awareness in this patient population, a population captured by the recently proposed term ‘cognitive motor dissociation’ (CMD) [7] is significant, because it fundamentally changes their capacity for well-being. There is widespread agreement in the philosophy of well-being that an important constituent of well-being is the experience of pleasure, and avoidance of pain. However, it is unclear if CMD patients can experience pleasure, or pain. In this paper, I argue that we have good reason to think that these patients can experience pleasure and pain. Accordingly, we have good reason for taking steps to promote their experience of pleasure, and reduce their experience of pain, thereby promoting their well-being.

## Unresponsive Wakefulness Syndrome: Background

According to the Multi-Society Task Force on PVS (Persistent Vegetative State), individuals with UWS/VS “demonstrate no evidence of awareness of self or environment and an inability to interact with others; no evidence of sustained reproducible, purposeful or voluntary behavioural responses to visual, auditory, tactile or noxious stimuli; and no evidence of language comprehension or expression” [2]. A diagnosis of UWS/VS is made through a behavioural examination at the bedside, using an assessment tool such as the Coma Recovery Scale-Revised [8]. UWS/VS may be a relatively brief transitory state; patients may move from coma —a state of unarousable unconsciousness— [9], to a vegetative state, to the ‘minimally conscious state’ (MCS), —characterized by inconsistent but reproducible behavioural response to stimuli, and which can itself be sub-divided into MCS- and MCS+, based on the level of complexity of behavioural responses [10, 11]— and in some cases, make a good recovery. The longer a patient is in UWS/VS, however, the less likely they are to recover consciousness. The Multi-Society Task Force on PVS regards it as ‘permanent’ one year after traumatic brain injury, or three months after anoxic brain injury [2].

Accurately diagnosing UWS/VS is highly challenging, with several studies indicating that nearly 43% of patients diagnosed as such show signs of awareness after repeated examination [12]. Given the challenges of accurate diagnosis, precise epidemiological data is difficult to ascertain; the prevalence of UWS/VS at least six months in duration is estimated to be between 40 and 168 per million people in the United States, with between 5 and 25 new cases per million people each year [13].

However, research over the last decade also indicates that a minority of patients who consistently show no evidence of voluntary behavioural response to stimuli at the bedside are nevertheless ‘covertly aware’ [3–5]. Using functional neuroimaging to monitor patient brain activity, researchers have been able to detect the volitional activation of specific brain areas in 17–19% of patients diagnosed as UWS/VS [4, 6]. These patients are asked to imagine performing a specific task, such as playing tennis or navigating their home, for 30 s intervals when instructed to do so by researchers, thereby activating specific brain areas. Following these 30 s intervals of activity, patients are instructed to ‘relax’, whereupon brain activity in the previously activated areas ceases. This provides robust evidence that patients understand the instructions they are given and are purposefully activating these brain areas, thus demonstrating their awareness. In fact, some patients have successfully used mental imagery to communicate with researchers, by providing correct ‘yes-or-no’ responses to autobiographical questions (e.g., ‘Is your name John?’ ‘Is your name Steve?’ ‘Are you in a hospital?’ ‘Are you in a grocery store?’) [14].

## Prudential Interests: Pleasure and Pain

One of the reasons that the discovery of awareness in these patients is important is because of its implications for their well-being. Well-being is concerned with prudential value, —of what is non-instrumentally, fundamentally good for a person— and encapsulates how well a person’s life is going for them. Different philosophical theories of well-being have different conceptions of what ultimately is prudentially valuable. However, it is a common feature of theories of well-being that they are concerned with what is good not impersonally, but for the person whose life it is [15]. When we think about what has prudential value for us, we do so from a subjective point of view. Because CMD patients have a subjective point of view, they satisfy a general requirement for having prudential interests.

Recognizing their capacity for prudential interests is an important step to ensuring that the well-being of these patients is protected. However, it is not obvious what the prudential interests of these patients are. While successful completion of the mental imagery task illuminates some of their residual cognitive capacities, we are still left with questions about how these patients experience the world. Indeed, prior to the discovery of covert awareness, they were presumed to be incapable of any experience at all. Given their injuries, it seems likely that many of the things that make our lives go well as healthy adults will be inaccessible to them. Practically speaking, promoting the well-being of these patients must focus on prudential goods which are both valuable to them, and that they can realize. One place we might begin is with the experience of pleasure and pain. If CMD patients can experience pleasure and pain —if they are ‘sentient’— promoting their experience of this prudential good (and minimizing their experience of this ‘prudential ill’) could be an effective means of promoting their overall well-being. Yet, as we saw earlier, UWS/VS is defined by the absence of behavioural response to stimuli; even if a minority of patients diagnosed as UWS/VS are covertly aware, it is not clear that they can experience pleasure, or pain.

In a philosophical context, the term ‘sentience’ is typically used in either a broad or narrow sense. An organism is sentient in the broader sense if it has the capacity for subjective experiences, when there is ‘something it is like’ to be that organism. Alternatively, an organism is sentient in the narrower sense when it has the capacity for subjective experiences of a certain affective quality, such as pain, pleasure, suffering, or enjoyment. Sentience in the narrower sense requires sentience in the broader sense, but not the converse. The purpose of this paper is to show that CMD patients are sentient in the narrower sense.

Why begin by considering pleasure and pain, and not some other prudential interest? First, there is a consensus both within the philosophy of well-being, and, I would suggest, our common-sense intuitions, that pleasure and pain are critical components of well-being. The experience of pleasure tends to make us better off, and the experience of pain tends to make us worse off. While not impossible, it is hard to imagine a person’s life going very well if they are in constant pain, or have very few pleasant experiences.

Second, focussing on the experiences of pleasure and pain makes sense from a pragmatic standpoint. Whereas there may be other prudential interests the satisfaction of which might promote patient well-being —such as the development of deep personal relationships, or the achievement of one’s goals— these are likely beyond the capacity of caregivers to satisfy. Conversely, if a patient is in pain, and can communicate this to a caregiver, concrete measures can be taken to reduce or eliminate the pain. Similarly, caregivers can provide pleasurable stimuli, (e.g., music, television, social interaction) to promote patient well-being.

Because pain and pleasure are subjective experiences, the only way we can verify that someone is having such an experience is via their self-report. For those patients in whom communication via mental imagery is not possible, then, we cannot directly verify their capacity for pain or pleasure. Nevertheless, as I argue in this paper, there is substantial empirical evidence to support the hypothesis that CMD patients are sentient in the narrow sense that is relevant to well-being. Moreover, even in the absence of conclusive evidence of sentience, I argue that we ought to treat CMD patients as if they are sentient. My argument will proceed as follows. In Part 1, I will argue that if CMD patients are sentient, then their interests in avoiding pain and experiencing pleasure matter morally. In Part 2, I will present evidence in support of the hypothesis that CMD patients can experience pain. In Part 3, I will do the same for pleasure. In Part 4, I will argue that even in the absence of certainty about the sentience of CMD patients, we ought to treat them as if they are sentient. Finally, in Part 5, I will discuss how caregivers might promote the well-being of these patients by promoting pleasure, and preventing pain.

## Part 1: Consideration of Interests and Well-Being in CMD Patients

Suppose CMD patients are in fact sentient. How should this affect our treatment of them? One possible response is that we should do what we can to minimize any suffering they experience, including treating their physical pain, and making reasonable efforts to promote their pleasure. The reason for this is that sentient interests (i.e., the interest that sentient beings have in avoiding pain and experiencing pleasure) matter morally; we have a reason to avoid frustrating these interests, and that reason exists simply in virtue of the harm frustrating these interests causes to the being that has them.

I have an interest in avoiding suffering, because my suffering is bad or harmful for me; others have an interest in avoiding suffering, because their suffering is bad for them. If I take the badness of my suffering for me to be a reason for others not to cause me to suffer, the badness of others’ suffering for them must give me reason to avoid causing them to suffer. In other words, I cannot disregard their interest in avoiding suffering, just because it is not *my* interest; their interests matter morally. Thus, insofar as CMD patients are sentient, their sentient interests matter morally. As a result, we have a reason to avoid causing these patients to suffer (or allow them to suffer when we could easily prevent this), and we would need sufficiently strong moral reasons opposing the alleviation of their suffering to justify failing to do so.

Debates about the treatment of patients with disorders of consciousness, including CMD patients, often place a great deal of weight on whether or not we should consider these patients to be ‘persons’ (i.e., whether their interests matter morally to the same degree as ‘moral persons’ like healthy adult humans) [16–18]. These debates often turn on disagreements about the necessary and sufficient conditions for personhood, the moral significance of these particular conditions, as well as the rights and obligations which personhood confers. For the purposes of my argument, however, it is not necessary to establish whether or not CMD patients are persons. The fact that these patients are sentient is enough to motivate an obligation to reduce their suffering, whether they are persons or not.^1^

In the next two sections, I will show that there is considerable empirical evidence to support the notion that CMD patients are sentient.

## Part 2: Pain

The International Association for the Study of Pain (IASP) defines pain as: “an unpleasant sensory and emotional experience associated with actual or potential tissue damage, or described in terms of such damage” [19]. As reflected in this definition, there seem to be two important components to our common-sense understanding of pain. On the one hand, pain has a sensory component. We can describe various sensory aspects of our painful experience, such as its intensity, its duration, and its (general) location. When we say that we are in pain, we are reporting on this perceptual experience.

On the other hand, pain also seems to have an affective aspect. A painful experience feels unpleasant, or aversive. Moreover, our experience of pain seems to be private, subjective, and about which we cannot be mistaken. It is private insofar as no one can access my pain in the way that I can (i.e., by feeling it). It is subjective in that it depends for its existence on my feeling it. Finally, if I feel that I am in pain, or believe that I am in pain, then I am in pain. Understood in this way, rather than being *objects* of perceptual experience, pains just are experiences *themselves*. And, unlike other perceptual experiences, which are liable to error (i.e., because my perception of an object is different from the way the object really is), the ‘true’ nature of pain just is the experience of it [20].

**Fig. 1:**
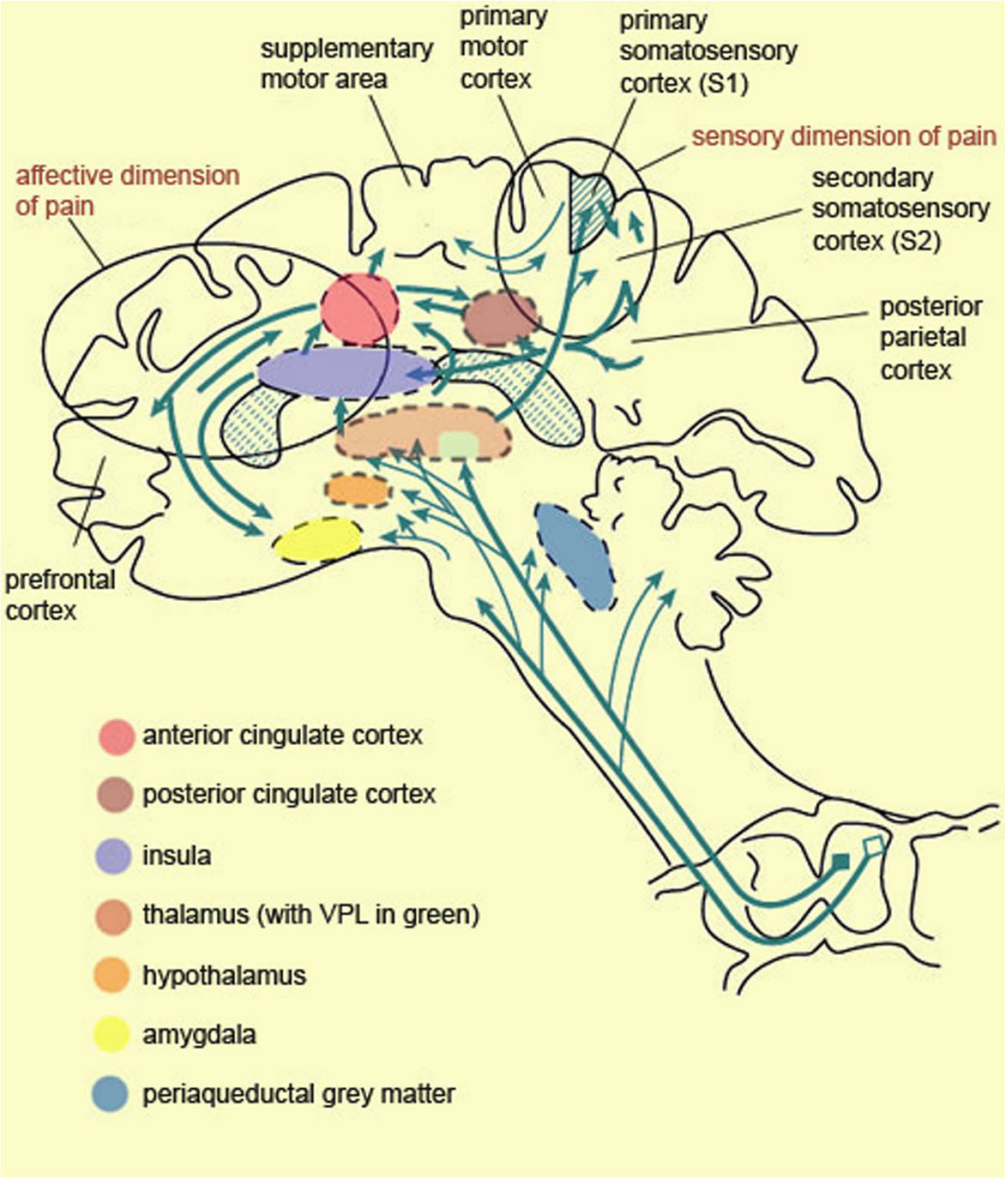
The Pain Matrix [22]

This conception of pain as a multi-dimensional experience is supported by neurophysiological evidence. The classic view of our basic pain system comprises two largely segregated subsystems, referred to as the ‘pain matrix’: the lateral neuronal network and the medial neuronal network [21]. These networks correspond to the sensory-discriminative and affective-motivational dimensions of pain (Fig. 1).

The lateral neuronal network encodes sensory-discriminative information, and generates an individual’s sense of the location, intensity, duration, and nature of painful stimuli. This network is composed of nociceptors called A-delta fibres, myelinated neurons which transmit information rapidly along the network from the spinal column, to the midbrain (i.e., periaqueductal matter) and ventroposterior lateral nucleus of the thalamus [23]. From there, nociceptive information is transmitted to the primary (S1) and the secondary somatosensory (S2) cortex, as well as the posterior insula [23]. These nociceptors transmit what is known as ‘fast pain’, and remain active as long as nerve endings are stimulated.

The medial network encodes the affective-motivational dimension; this corresponds to the feeling of aversion that typically accompanies the physical sensation, and the urge to avoid or withdraw from the stimuli perceived as responsible for the disliked feeling. This network is composed of nociceptors called C-fibers, unmyelinated neurons which transmit information more slowly than A-delta fibers, and thus are responsible for what is referred to as ‘slow pain.’ These nociceptors will continue to fire for a period of time after noxious stimulation has ceased, and transmit information to the anterior insula, the anterior cingulate cortex (ACC) and the prefrontal cortex, as well as the posterior cingulate cortex [24].

While these networks typically are activated together (i.e., a pain experience usually has both a sensory and affective-motivational dimension), this is not always the case. Indeed, a great deal of empirical research supports the notion that the sensory-discriminative and affective-motivational dimensions of pain can be dissociated [25, 26]. The fact that the sensory and affective-motivational aspects of pain experience are only contingently related allows us to explain cases in which individuals report that they feel ‘pain’ but do not mind it; they are experiencing the sensory aspect of pain, but not the affective-motivational aspect. Similarly, individuals who seem to enjoy pain (e.g., masochists) may be having the same sensory experience as non-masochists when subject to a certain stimulus, but whereas these sensations arouse in non-masochists feelings of aversion, they arouse in masochists a different collection of motivations or affect.

There has been considerable discussion in the philosophical literature concerning the nature of pain, and whether it is essentially a sensation, an affective experience, or something else [27]. For present purposes, however, these issues can be largely set aside. Whether we take the essence of pain to be its sensory quality, our affective-motivational response, or some combination of the two, it seems clear that it is our affective-motivational response to painful stimuli that is responsible for our feelings of *suffering* because of physical pain. Whatever ‘pain’ turns out to be, the experience of physical pain causes us to suffer only insofar as we have the appropriate aversive response to it; this is the role of the affective-motivational dimension of pain. And, it is suffering that is most relevant to our well-being.

Therefore, if CMD patients experience the affective-motivational dimension of pain (i.e., they experience the feeling of aversion characteristic of a physically painful experience), it follows that they are capable of suffering, and thus, of having their prudential interests frustrated. While we cannot assess this directly in the absence of direct communication, we can use activity in the medial neuronal network as a proxy for a patient’s experience of the affective-motivational dimension of pain. Thus, while activity in the medial network does not prove that these patients are experiencing the affective-motivational dimension of pain, it nevertheless provides good evidence that they are [28–30].

## Evidence for Pain Experience in Patients Diagnosed as UWS/VS

UWS/VS patients will occasionally produce motor or autonomic responses to painful stimuli (e.g., changes in respiratory frequency, increased heart rate and blood pressure, changes in pupillary diameter, increased skin conductance) [31]. Similarly, presentation of a noxious stimulus may elicit involuntary decerebrate (abnormal extension of the arms by the side), or decorticate (flexion of the arms, elbows, wrists and fingers, inward towards the chest) posturing in coma patients, as well as UWS/VS patients [2]. However, because these responses can occur reflexively (i.e., in the absence of conscious awareness), they are not reliable evidence of genuine pain experience, with the affective component that entails [32].

Several studies have attempted to investigate the possibility that UWS/VS patients can experience pain. Laureys and colleagues used positron emission tomography (PET) imaging to measure cerebral metabolism in 15 UWS/VS patients when presented with high-intensity electrical stimulation of the median nerve, and compared this to the cerebral metabolism of 15 healthy controls [33]. While they found that overall cerebral metabolism was 40% of normal values in UWS/VS patients, they detected activation in the midbrain, contralateral thalamus, and primary somatosensory cortex in each of the UWS/VS patients. However, they found that noxious stimulation elicited no downstream activation beyond the primary somatosensory cortex. Moreover, functional connectivity assessment showed that the observed cortical activity was functionally disconnected from higher order associative cortices (i.e., secondary somatosensory, bilateral posterior parietal, premotor, polysensory superior temporal, and prefrontal cortices) that are currently thought to be necessary for conscious awareness. This suggests that despite the observed brain response to noxious stimulation, it is unlikely that these patients retain the capacity to consciously experience pain.

Similarly, Kassubeck and colleagues used positron emission tomography to analyze central processing of pain using electrical stimulation in 7 anoxic UWS/VS patients [34]. Like Laureys and colleagues [33], they found activation in the primary somatosensory cortex—as well as in the secondary somatosensory cortex, insular, and anterior cingulate cortices—but found these areas to be dissociated, with no functional connectivity between higher order associative cortices [34]. A 2005 study by Boly and colleagues also found that while many brain regions of the pain network are activated in UWS/VS patients, they are disconnected from each other [35]. While UWS/VS patients may retain islands of cortical functioning within areas of the brain involved in pain experience, the functional disconnection between these areas makes it unlikely that patients are consciously aware of the noxious stimuli, and thus, unlikely that they are experienced as aversive [36].

These results are supported by recent findings which suggest that UWS/VS patients present a severe impairment of backward connectivity in higher-order associative cortices, which is crucial for the sort of integrated brain processing necessary for consciousness, and thus, the conscious experience of pain. Boly and colleagues measured effective connectivity in backward and forward connections at two hierarchical cortical levels (i.e., temporal and frontal cortices) in UWS/VS and MCS patients, as well as healthy controls, during auditory processing [37]. They found that UWS/VS patients exhibited significantly impaired backward connection from frontal to temporal cortices compared to both MCS and controls. They concluded that recursive processing in high-order cortical areas is necessary for the generation of conscious perception. Thus, because primary cortical activation is isolated from higher-order associative cortical activity in patients in the vegetative state, these patients are highly unlikely to be capable of conscious experience, including the conscious experience of pain [37]. Similarly, Laureys and colleagues point out that in rare cases where UWS/VS patients recover awareness, PET shows a functional recovery of metabolism in the associative cortices—bilateral prefrontal regions, parieto-temporal, and posterior parietal areas—as well as functional connectivity within these areas [38].

## Pain Experience in CMD Patients

What reason do we have for thinking that CMD patients can experience pain? First and foremost is the fact that they satisfy a necessary condition for experiencing pain: they are aware. While awareness does not entail sentience, the absence of awareness eliminates the possibility of sentience. Thus, the discovery of awareness in CMD patients removes a significant barrier to sentience.

Second, there is evidence to suggest that some patients diagnosed as UWS/VS may nevertheless retain activity within the neural structures typically involved in the conscious experience of pain. As mentioned above, Kassubeck and colleagues found pain-induced activation in a broad pain-related cerebral network, including areas associated with the affective dimension of pain (i.e., secondary somatosensory cortex, insular, and anterior cingulate cortices) in seven anoxic VS patients [34]. Moreover, in a 2013 study, Markl and colleagues used fMRI to investigate brain activation in response to noxious electrical stimulation of the right index finger in 30 patients diagnosed as UWS/VS [39]. While no pain-related activation was found in 14 patients, 15 patients diagnosed as UWS/VS showed activation in the sensory-discriminative part of the pain matrix (S1, S2, posterior insula, thalamus), and nine patients showed activation in the affective part (anterior cingulate cortex, anterior insula). About one-third of the patients had pain-related responses in both the sensory and affective-motivational parts of the pain matrix. While neither of these studies demonstrates definitively that patients diagnosed as UWS/VS experience pain (insofar as activation within a certain brain area cannot prove the presence of a certain subjective experience), they do suggest that brain activation in response to noxious stimuli may be more extensive in these patients than initially thought, leading some to speculate that patients with activation of the affective pain network may experience pain [40].

Third, the functional neuroimaging studies cited above [33, 35] strongly suggest that the reason UWS/VS patients are incapable of experiencing pain is because the neural structures involved in the conscious processing of pain (i.e., the pain matrix) are either inactive, or functionally dissociated from one another. In short, these patients may experience something like painful sensations, but due to a lack of connectivity within the pain matrix, lack the affective component of pain which makes it unpleasant, and thus, concerning for their welfare. Accordingly, if the connectivity between these brain areas was present in CMD patients, this might give us further reason to believe that these patients can experience pain.

There is good reason for thinking that CMD patients may retain functional integrity of the pain matrix. Intact functional connectivity between primary and associative cortices appears to be a critical component of conscious awareness. Thus, it seems reasonable to suppose that CMD patients —patients that have demonstrated that they retain a degree of functional connectivity sufficient to allow for conscious awareness— may also retain functional connectivity of the pain matrix. Moreover, the patients under consideration here have demonstrated significant cognitive ability in preforming the mental imagery task, including attention, working memory, and language comprehension [14]. Sentience, on the other hand, is thought to be a much more basic element of consciousness [41], which supports the view that if CMD patients can perform the complex cognitive tasks necessary to complete the mental imagery task, they likely retain the capacity to consciously experience pain.

Consider another group of patients with disorders of consciousness, who are thought to be capable of pain experience: patients in the minimally conscious state (MCS) 11]. Studies involving these patients have produced a great deal of evidence, both behavioural and through functional neuroimaging, to support the idea that they can experience pain [42]. For example, Boly and colleagues studied brain activation induced by bilateral electrical stimulation of the median nerve in five MCS patients, and found activation in the thalamus, primary somatosensory cortex, the secondary somatosensory, as well as the frontoparietal and anterior cingulate cortices (i.e., the ‘pain matrix’) [43]. No area was less activated in MCS patients than healthy controls. In contrast to UWS/VS patients, MCS patients displayed preserved functional connectivity between the thalamus, primary somatosensory cortex, and a wide cortical network, including the secondary somatosensory cortex, the posterior cingulate cortex, and the anterior cingulate cortex. Moreover, patients in MCS have been shown to retain larger cortical activation than UWS/VS patients using other modes of stimulation, and demonstrate a better connectivity between the primary and associative cortices [38, 43–45].

The results of these functional neuroimaging studies are confirmed using behavioural scales in MCS patients. The Nociception Coma Scale-Revised is used to assess nociception in non-communicative patients recovering from coma (i.e., UWS/VS and MCS patients), by measuring behaviours believed to be indicative of nociception [46]. It has been shown to have a strong correlation with other validated pain scales, as well as sensitivity to the difference between VS and MCS patients, which suggests that it is assessing pain. Schnakers and colleagues used this scale to measure the responses of 28 VS patients and 20 MCS patients to the application of pressure to the fingernail bed [47]. Both groups of patients displayed responses to the noxious stimulation; however, the scores obtained were higher in MCS patients than VS patients. While VS patients exhibited stereotypical responses linked to brainstem activation, (e.g., startle response, abnormal flexion like decerberate or decorticate posturing), MCS patients exhibited responses linked to subcortical and cortical activation (e.g., flexion withdrawal from painful stimulus, purposeful movements directed at the site of the noxious stimulation, and visual fixation). Furthermore, a study by Chatelle and colleagues has demonstrated a significant positive correlation between scores on the Nociception Coma Scale Revised and activity in the anterior cingulate cortex, the cerebral area which most consistently displays activation during the experience of pain [48].

The fact that MCS patients retain functional connectivity of the pain matrix is highly suggestive of a connection between the presence of awareness, and the ability to consciously experience pain. Because the functional integrity of the pain matrix (i.e., primary somatosensory and associative cortices) is good evidence of the experience of pain, and because conscious awareness appears to require a high degree of cortical integration (i.e., functional connectivity between thalamocortical areas and cortico-cortical areas) [37, 38], it is likely that CMD patients retain functional integrity of the pain matrix, and thus, the capacity to consciously experience pain [49].

A further concern is those patients in whom awareness has not been detected, but in whom we cannot be certain that awareness is absent. Indeed, it may be the case that a subset of patients who have not demonstrated covert awareness are nevertheless aware, and thus, may retain functional connectivity of the pain matrix. Moreover, given the high rate of misdiagnosis of patients in the vegetative state [12], it is not unreasonable to suppose that some patients behaviourally diagnosed as vegetative may nevertheless be aware, and perhaps sentient. Indeed, the studies mentioned above by Kassubeck and Markl [34, 39] show that some patients believed to be vegetative show activation within both the sensory and affective dimensions of the pain matrix; it might be the case that these are patients in whom awareness is present, but remains undetected. However, consideration of this broader patient population is outside the scope of this paper.

## Part 3: Pleasure

What exactly is pleasure? On a common-sense understanding, it is a feature of experience that makes those experiences good and attractive. In some cases, the experience or expectation of pleasure can motivate us to pursue certain rewards, or otherwise shape our behavior. As we saw earlier when considering pain, there is a rich philosophical literature concerning the nature of pleasure [50]. I will not enter this debate here. My interest is determining whether non-communicative patients with covert awareness can experience pleasure as it is generally understood, that is, as a subjective feeling of niceness, liking, or positive affect.

Research in affective neuroscience suggests that the experience of pleasure consists of an interacting, yet distinguishable, set of processes, each with distinct underlying neurobiological mechanisms. As Berridge and Kringelbach describe, a pleasurable experience, or ‘reward’, consists of three components: ‘liking’, ‘wanting’, and ‘learning’ [51]. ‘Liking’ is the actual pleasure component, or hedonic impact of a reward; ‘wanting’ is the motivation for reward, including conscious desiring and unconscious incentive salience; and ‘learning’ is the associations and predictions we make based on past reward experiences. When we experience pleasure, it is because a certain stimulus has activated those neurobiological mechanisms.

In most cases, ‘liking’ and ‘wanting’ work in tandem. However, these processes result from distinct brain mechanisms, and can be dissociated. For example, research has shown that rats with extensive damage to dopamine receptor neurons, or pharmacological dopamine blockade, fail to approach (i.e., ‘want’) sweet foods but nevertheless exhibit normal hedonic ‘liking’ reactions when the food is placed in their mouths [52]. These rats do not attribute incentive salience to rewards —they do not eat when surrounded by appetizing food— yet they consume as much as normal rats when forced to eat, and show signs of hedonic liking during consumption. Conversely, mice with elevated dopamine levels appear to more aggressively pursue rewards (suggesting increased ‘wanting’) without exhibiting higher orofacial ‘liking’ activity. Moreover, studies in humans have shown that dopamine levels are more highly correlated with subjective ratings of wanting a reward, than with subjective ratings of liking of the same reward. [52] Indeed, highly addictive drugs are an example of a stimulus being highly ‘wanted’, while producing little to no feelings of ‘liking’ in the user [53].

How is this feeling of ‘liking’ produced in the brain? As Frijda argues, pleasure itself is not a sensation; rather, it is a ‘pleasantness gloss’ added to stimuli that the brain identifies as pleasant [54]. Thus, brain signals representing mere sensations are transformed into hedonic stimuli, by coincident hedonic neural activity that imbues them with pleasure. As Kringelbach argues, whether a given stimulus is experienced as pleasurable (i.e., whether it elicits a positive hedonic signal, or generates a ‘liking’ reaction in the brain), depends on several factors, including motivation-independent processing of identity and intensity; formation of learning-dependent multimodal sensory representations; reward representations using state-dependent mechanisms including selective satiation; and representations of hedonic experience, learning, or direct behavioral change [55].

As Berridge and Kringelbach argue, liking reactions to pleasure-inducing stimuli can be both conscious and non-conscious [53]. At the non-conscious level, ‘liking’ is an objective hedonic reaction, generated by specific brain systems, and may occur without an accompanying subjective feeling of pleasure. Just as unconscious visual processes may be translated into visual sensations, non-conscious ‘liking’ reactions are ordinarily translated into conscious pleasure feelings by additional cognitive brain mechanisms. Thus, non-conscious ‘liking’ is a component of conscious liking in pleasure, but may occur independently as merely a non-conscious ‘liking’ reaction, which can be measured behaviourally or neurally. Non-conscious ‘liking’ reactions still effectively change goal-directed human behavior, though these changes may remain undetected or misinterpreted by the individual having them. At the conscious level, liking is the conscious experience of pleasure in the ordinary sense of the word, and is experienced subjectively [46, 52, 53].

Much of the work that has been done investigating how pleasure is generated in the brain has appealed to brain areas which generate objective ‘liking’ reactions, referred to as ‘hedonic hotspots’ [51]. Using microinjections at various sites of the brain to stimulate pleasure-generating systems in rodents, researchers have been able to identify where neurochemical signals generate a ‘liking’ reaction. These studies have revealed an interconnected network of hedonic hotspots, mostly located in subcortical structures, including the nucleus accumbens, ventral pallidum, and the brainstem. This network forms a functionally integrated circuit, with activity in one hot spot recruiting activation across other hotspots simultaneously, with greater breadth of activation resulting in greater production of pleasure, unless another hotspot suppresses activity. In fact, the available evidence suggests that brain mechanisms involved in basic sensory pleasures (e.g. food, sex), overlap to a high degree with ‘higher order’ pleasures (e.g., artistic, monetary, altruistic, social) [51].

While neurochemical activation of this network of hedonic hotspots can significantly amplify the experience of pleasure, only some of these hotspots are also necessary for maintaining normal hedonic levels of liking to pleasant sensations. In fact, normal liking reactions to pleasure are relatively difficult to abolish by any single event (e.g., a drug, or brain lesion) [51]. The most crucial hedonic hotspot appears to be in the ventral pallidum; research in rodents has shown that this hotspot is the only one where lesion damage results in the loss of normal sensory pleasure [51, 56]. Similarly, humans with ventral pallidum lesions have been reported to have ‘anhedonia’, a condition in which one has a reduced or absent ability to experience pleasure [57]. Conversely, studies have shown that while activation in the nucleus accumbens is sufficient to cause enhanced pleasure reactions, damage to this area may only subtly impair the hedonic impact of rewards [51]. Similarly, studies in both animal models and anencephalic infants suggests that in the absence of functional hedonic hotspots, the brainstem can generate rudimentary forms of affective reactions to pleasurable stimuli [56].

In contrast to areas which are necessary or sufficient *causes* of pleasure, other brain areas have been implicated in the *coding* of pleasure; activation in these areas is correlated with increases or decreases in pleasure, but not conclusively shown to be necessary or sufficient for pleasure. Neural coding of pleasure appears to occur in several brain sites, including the orbitofrontal cortex, anterior cingulate cortex, insular cortex, amygdala, nucleus accumbens, and ventral pallidum [51]. Information from hedonic systems is coded in the anterior parts of the orbitofrontal cortex, where it can be used to influence subsequent behavior (in lateral parts of the anterior orbitofrontal cortex with connections to anterior cingulate cortex), stored for valence learning and memory (in medial parts of the anterior orbitofrontal cortex), and made available for subjective hedonic experience (in mid-anterior orbitofrontal cortex) [51]. Activity in the mind-anterior sub-region of the orbitofrontal cortex has been shown to correlate strongly with subjective pleasantness ratings of various rewards, as well as tracking changes in subjective pleasure. However, because activity in these areas is merely correlated with pleasure, it may reflect pleasure causation or brain activity caused by pleasure, which in turn may cause another function elsewhere (e.g., memory of reward, or cognitive appraisal of reward) [51].

## Pleasure and Well-Being

Can non-conscious liking contribute to well-being? Given traditional philosophical accounts of well-being, the notion of a non-conscious pleasure is somewhat awkward. Hedonist accounts of well-being argue that pleasure is intrinsically good (and pain intrinsically bad), but these accounts seem to imply the *experience* of pleasure. Pleasure is good because of how it feels, or because of our conscious attitudes with respect to pleasant things; it is unclear how we could enjoy the feeling of a non-conscious pleasure. Desire-satisfaction accounts of well-being argue that prudential value consists in having our desires satisfied. While non-conscious pleasures might be desirable because of how they color our other experiences, it is hard to see how they might be desirable in and of themselves. Similarly, objective-list views of well-being claim that certain goods are intrinsically valuable, independently of our attitudes towards them. While some objective-list theories hold that pleasure is an objective good, one would assume that this means pleasure that is subjectively experienced.

Nevertheless, even if non-conscious liking is not intrinsically valuable, it may still positively contribute to well-being. Indeed, non-conscious liking reactions can influence our behavior and motivations, while remaining undetected by the person having them. For example, Berridge and Winkeilman suggest that it is possible to generate an affective reaction, of which the individual is not consciously aware [58]. In their experiment, researchers presented participants with subliminal exposures of happy, neutral, or angry faces, and then asked participants to rank their subjective emotion at that moment on a 10-point scale (from ‘very unpleasant’ to ‘very pleasant’). Following this, participants were presented with a pitcher of fruit-flavored drink, and asked to pour and consume as much as they wanted, and evaluate it. They found that participants exposed to happy faces poured and consumed roughly 50% more of the drink than those who had seen neutral faces. Importantly, none of the participants reported any change in their subjective emotion ratings. The researchers concluded that participants’ affective state had been positively influenced by the subliminal presentation of the smiling face, which was reflected in their increased liking of the fruit-drink, without the participants being consciously aware of any change in their affective state.

In the case of CMD patients, non-conscious liking reactions may promote more positive moods, or help to mitigate the impact of negative moods or emotions, through the release of endogenous opioids. Endogenous opioids are involved in the non-conscious liking reactions to pleasant stimuli, and have been linked to an increase in pain tolerance when elicited by pleasant stimuli [59]. Similarly, several studies have measured the impact of mood on pain sensitivity, and found that using pleasant stimuli to improve mood can reduce pain perception [60]. Thus, even if these patients do not experience pleasure as a subjective feeling, providing them with pleasant stimuli may indirectly promote their well-being.

On the other hand, it seems uncontroversial that the conscious experience of pleasure would promote the well-being of CMD patients. In fact, it is somewhat surprising that there have been no studies investigating the capacity of these patients to experience pleasure. Perhaps this is because the experience of pain is thought to be more harmful to these patients’ well-being than the experience of pleasure is beneficial. In any case, if CMD patients can experience pleasure, providing them with pleasant experiences whenever possible seems an obvious way of promoting their well-being.

## Pleasure in CMD Patients

In the previous section, I considered the potential contribution of non-conscious and conscious pleasure to the well-being of CMD patients. I now turn to the issue of whether CMD patients are capable of either the non-conscious or conscious aspects of pleasure. Given that much of this patient population is non-communicative, and thus, incapable of self-report, how can we determine their capacity for pleasure experience? We have seen that several brain areas —hedonic hotspots— are either necessary or sufficient for the causation of objective ‘liking’ reactions. Thus, if these brain areas remain functionally intact in CMD patients, this would provide evidence that these patients remain capable of generating hedonic liking reactions.

There is evidence to suggest that this might be the case. Research investigating the relationship between clinical assessments of consciousness and underlying brain damage has shown that when compared to healthy volunteers, both traumatic and non-traumatic minimally conscious and vegetative patients show atrophy in the basal forebrain, which contains subcortical brain structures associated with pleasure causation, including the nucleus accumbens, and the ventral pallidum [61]. However, this study also found that arousal was inversely correlated with the degree of atrophy in the bilateral basal ganglia (the location of hedonic hotspots ventral pallidum and nucleus accumbens). This finding is consistent with literature which suggests that the basal ganglia serves a critical role in arousal, and regulating sleep-wake behaviour [62, 63]. Studies using positron emission tomography (PET) have demonstrated that when patients recover from coma to vegetative or minimally conscious state, they recover function in the basal forebrain, which in turn explains their recovery of sustained spontaneous eye-opening [64].

Accordingly, if damage to the basal forebrain is not sufficient to abolish wakefulness in CMD patients, it may also be the case that the brain mechanisms underlying pleasure causation in the basal forebrain are preserved as well. This hypothesis is supported by research linking the circuit of the basal ganglia, thalamus, and frontal cortex in supporting awareness. Several studies have shown that disruptions to this network leads to severe deficits in awareness [65, 66], while preserved functional connectivity of this network has been shown to accurately discriminate between patients with disorders of consciousness and healthy controls [67, 68]. While preserved function of the basal ganglia does not entail that the ventral pallidum continues to function in generating non-conscious liking reactions as it would in a healthy brain, it is suggestive of this fact. Further research is required to determine whether CMD patients continue to generate non-conscious liking reactions, either through the ventral pallidum, or other hedonic hotspots.

Conversely, while the brain areas which cause non-conscious ‘liking’ reactions to pleasurable stimuli are concentrated in subcortical structures, those which appear to support the conscious experience of pleasure are primarily in the orbitofrontal cortex. Given the extensive cortical damage of many CMD patients, we might be skeptical about their preserved capacity for subjective pleasure experience.

Firstly, although neuroimaging evidence clearly suggests that the orbitofrontal cortex codes for pleasure, it remains unclear if this area causes the consciousness of pleasure, more basic hedonic ‘liking’ reactions to pleasure, or whether it is a point of integration between non-conscious and conscious hedonic processing for decision making [51, 69]. The fact that individuals with prefrontal lesions (e.g., patients who have undergone prefrontal lobotomy) typically retain the capacity for basic ‘liking’ reactions suggests that the orbitofrontal cortex might be more important to translating hedonic information in cortical representations, than generating ‘liking’ reactions. Similarly, patients with certain types of clinical anhedonia —the inability to experience pleasure— still give normal hedonic ratings to many sensory pleasures. Shewmon and colleagues describe cases of hydrocephalic children with significant loss of cerebral tissue, who nevertheless expressed pleasure by smiling and laughing [70]. This suggests that the disruption of cortical activation patterns in orbitofrontal, insular, and other limbic regions which characterizes anhedonia may impair cognitive evaluations of pleasurable stimuli, without extinguishing the more basic capacity for sensory pleasure [69, 71].

Second, as Berridge and Kringelbach suggest, non-conscious ‘liking’ reactions “are ordinarily translated into conscious pleasure feelings by additional cognitive brain mechanisms that underlie subjective awareness” [51]. Indeed, it is the ability to convert a ‘liking’ reaction into a subjective experience of pleasure that may differentiate human conscious experience of pleasure from other animals. The fact that CMD patients remain conscious implies that they at least retain a necessary requirement for subjective pleasure experience. Moreover, research has shown that connectivity within the default mode network is closely correlated to the level of consciousness of brain damaged patients [72]. This network of brain regions has also been speculated to be involved in the subjective experience of pleasure [71].

The default mode network is a set of brain areas —including the precuneus, bilateral temporo-parietal junctions, and medial prefrontal cortex— which has been found to be more active at rest than when subjects are preforming attention-demanding cognitive tasks. This network has been demonstrated to be involved in cognitive process like daydreaming, stimulus-independent thoughts, and self-related thoughts, and has been suggested as a network supporting consciousness [73, 74].

In a study by Vanhaudenhuyse and colleagues, default mode network connectivity strength was found to be proportional to the level of consciousness of brain damaged patients (including minimally conscious, vegetative, and coma patients) [72]. While the default mode network was still identifiable in unconscious patients, variation in the strength of default network connectivity (particularly the precuneus) was sufficient to differentiate between minimally conscious and unconscious patients, suggesting a particularly strong relationship between the level of activity of this area and the patients’ level of consciousness.

Given this relationship between connectivity of the default mode network and level of consciousness, we would expect that CMD patients would retain a degree of connectivity of the default mode network. If this is the case, it lends support to the idea that these patients may retain the capacity for the subjective experience of pleasure. Key regions of the frontal default mode network overlap with the brain areas implicated in the subjective experience of pleasure, such as the anterior cingulate and orbitofrontal cortices [71, 75]. Moreover, activity changes in the frontal default network, such as in the subgenual cingulate and orbitofrontal cortices, have been shown to correlate with pathological changes in subjective hedonic experience, such as in patients with depression [71, 76]. Further research is required to better understand the relationship between the default mode network and the subjective experience of pleasure. Moreover, depending on the extent of the damage to the brains of the patients under consideration, their subjective experience of pleasure —particularly cognitive aspects of pleasure experience— may be impaired, and thus, differ in some ways from the experience of healthy individuals. Nevertheless, this potential role for the default mode network in the subjective experience of pleasure lends further support to the notion that patients diagnosed as vegetative can experience pleasure.

## Part 4: Precautionary Reasoning for Treating CMD Patients as Sentient

As the above discussion shows, there is significant evidence to suggest that CMD patients can experience pain and pleasure. Acknowledging the fact that these individuals may still experience pains and pleasures of various kinds is critical to protecting their well-being; they are not simply ‘hedonically inert’, although they do not respond behaviourally to stimuli.

Nevertheless, while I think that the evidence in support of sentience is strong, it is not decisive; it is possible (though I would argue unlikely) that CMD patients are not sentient. Taking this lack of certainty into account, I argue that we ought to treat CMD patients as if they are sentient, and thus, that we should treat their putative sentient interests as mattering morally. In doing so, I appeal to the idea of ‘precautionary reasoning’. Roughly, precautionary reasoning holds that when there is a lack of conclusive evidence for X, preventative measures against X may be justified, provided the consequences of X are sufficiently adverse [77]. For example, if I am uncertain whether a dinner guest has a serious peanut allergy, I should avoid serving them food cooked with peanut oil.^2^

CMD patients can either be sentient, or not, and accordingly, their interests can either matter morally on those grounds, or fail to matter morally on those grounds. If they are sentient, it would be a large loss to mistakenly treat them as non-sentient (we would be harming them by failing to give their interests the consideration that they are owed), while it would be a large gain to correctly treat them as sentient (we would be giving their interests due consideration). Conversely, if a CMD patient were not sentient it would be a small gain to correctly treat them as non-sentient (we would avoid giving their interests greater consideration than we ought to), while it would be a small loss to mistakenly treat them as sentient (we would give their interests greater consideration than we ought to). In order to avoid the worst possible outcome (disregarding existing interests), and make possible the best possible outcome (regarding existing interests), we should treat CMD patients as if they are sentient.

What makes the use of precautionary reasoning appropriate in this case is the strong empirical evidence cited in the previous two sections in favor of sentience in CMD patients. Indeed, each of the four possibilities mentioned above are not equally likely; it is more likely the case that CMD patients are sentient than that they are not. Thus, the most likely set of outcomes is the large benefit or the large loss, rather than the small benefit and small loss. Therefore, even if we remain uncertain about the sentience of CMD patients, the preponderance of evidence, in conjunction with precautionary reasoning, strongly supports treating CMD patients as sentient.

A potential objection to applying precautionary reasoning to the sentience of CMD patients is that it ignores the potential costs of the outcome wherein a non-sentient patient is treated as sentient. Indeed, insofar as this unnecessary cost deprives other individuals of health-care resources, we might think that this is not merely a small loss, but a large one.

However, as I describe in Part 5, the kinds of interventions which could be useful in alleviating the potential suffering and promoting the well-being of CMD patients are not particularly burdensome; it is unlikely that the costs of providing these benefits would deprive other needy individuals of scarce health-care resources in any significant way. Indeed, it is important to bear in mind that taking the sentient interests of CMD patients into consideration does not entail that they are entitled to *any* sort of treatment that might benefit them. Another patient may have a stronger claim to limited health-care resources in virtue of the fact that they have more interests at stake (i.e., in addition to their interest in experiencing pleasure and avoiding pain). My claim here is that the sentient interests of CMD patients give us reason to alleviate their suffering, simply in virtue of the fact that their suffering is harmful to them. We might have competing moral reasons against alleviating their suffering, which may be stronger in some cases (e.g., if alleviating the suffering of one CMD patient meant failing to alleviate the suffering of 1000 children). However, given the fact that the cost of alleviating the suffering of CMD patients (by minimizing pain and promoting pleasure in the ways I describe below) is unlikely to cause any significant harm or loss of benefit to others, there is no competing moral reason sufficient to override our obligation to alleviate the suffering of these patients.

## Part 5: Promoting Pleasure and Minimizing Pain in CMD Patients

One complicating factor in alleviating the suffering of CMD patients is their inability to communicate the cause of their potential suffering. While it may be safe to assume that intense physical pain causes a patient to suffer, eliminating the source of suffering may cause the patient to suffer in other ways. For example, while administering a strong sedative may reduce the patient’s suffering caused by physical pain, it may cause greater suffering to the patient by reducing their level of awareness. However, it seems reasonable that when subjecting these patients to procedures known to cause physical pain, certain treatment measures (e.g., the provision of a mild analgesic such as acetaminophen) should be taken, because they are unlikely to cause additional suffering in the patient [78]. Further research is needed to develop evidence-based guidelines for the prevention and treatment of suffering in these patients, especially how the experience of physical pain may result in patient suffering.

The practice of pain assessment and management of non-communicative patients with dementia, in long-term care facilities, may offer guidance. Pain assessment in this population presents many of the same challenges as in CMD patients (e.g., inability of patients to report pain, patient interpretation of noxious stimuli influenced by cognitive impairments, difficulty in interpreting behaviors which may be indicative of pain) [79, 80]. Several methods for assessing pain in non-communicative patients with dementia have been developed, including care-giver observation and surrogate report [81–83]. A consensus statement outlining a hierarchy of pain assessment techniques for use in non-communicative patients has been developed by the American Society for Pain Management Nursing [84]. This statement recommends a detailed history and physical examination to determine potential causes of pain that may be affecting the patient; direct observation of the patient to identify behaviors activities, or interactions that may be suggestive of pain; appeal to surrogate reports to identify changes in behavior; and finally, response to a trial of low-dose analgesics. These guidelines might be adapted to the assessment of CMD patients. Importantly, qualitative research clearly supports the fact that close knowledge of the patient is extremely important in detection of pain in patients with dementia [81]. This suggests that the input of primary caregivers (i.e. nursing staff, patient’s family) is of the outmost importance in recognizing patient pain.

Similarly, acknowledging a patient’s capacity for pleasure may influence how this patient is cared for. Knowing that a patient can experience pleasure may vindicate the efforts of caregivers to provide them with pleasant experiences, and engage with them in other ways, even when the patient’s ability to respond behaviourally is absent (or limited). For example, one patient who had been behaviourally non-responsive for 16 years, but was subsequently found to be covertly aware [85] regularly attended the movies with his family [86]. Engaging in pleasant activities like this can help to enrich the lives of these patients, and the knowledge that their family members can find these experiences pleasant may provide confirmation to caregivers that their efforts to promote the well-being of these individuals are worthwhile.

If CMD patients are sentient, this ought to influence not only the stimuli they receive, but how these stimuli are provided. Numerous studies have shown that anticipating pain, or being anxious about pain, can exacerbate the pain experienced [87]. Similarly, studies of ‘catastrophizing’ in patients with fibromyalgia has shown that this response influences pain perception, by altering attention and anticipation, as well as heightening emotional responses to pain [88]. If CMD patients are cognizant that a care intervention is taking place, but uncertain about whether it will be painful, this might exacerbate the negative hedonic impact. Caregivers may be able to mitigate this by communicating with patients, and explaining to them the nature of the intervention taking place.

It is also well-established that the hedonic impact of a stimulus can be affected by factors like homeostatic balance; the pleasantness of a stimulus increases the more effective that stimulus is at restoring bodily homeostasis, while pain unpleasantness increases with greater perceived threat to homeostasis [59]. Excessive exposure to a previously pleasant stimulus may result in this stimulus becoming aversive; a first chocolate bar tastes pleasant, but a fourth, much less so. Because CMD patients cannot control their exposure to pleasant or painful stimuli, caregivers must be mindful of the possibility of putatively pleasant stimuli becoming aversive to patients, either due to excessive exposure, or due to other changes in a patient’s homeostatic balance.

## Conclusion

The presence of covert awareness in some patients diagnosed as vegetative implies that these patients have prudential interests. Yet, given our nascent understanding of the subjective experiences of these individuals, we might wonder what sorts of things have prudential value for them. I argue that a natural starting point is the experience of pleasure and pain. While it may turn out to be the case that some CMD patients do not experience pleasure (or pain), this would not undermine the argument I set out to make in this paper. The potential harm of ignoring a patient’s capacity for pain is considerable, whereas the potential harm of mistakenly assuming a capacity for pain is low. Similarly, providing a patient with pleasant stimuli may improve their overall well-being if they are capable of pleasure; if they are not, it results in no significant harm to the patient. For these reasons, we ought to treat these patients as capable of sentience, provided we have some evidence to suggest that they are indeed sentient. I have provided such evidence in this paper. By acknowledging the capacity of these patients for pleasure and pain, and moreover, acting to minimize their experience of pain and promote their experience of pleasure (such as in the ways I describe in this paper), caregivers and family members of these individuals can make a positive step in helping them to live lives of at least decent quality.

## Footnotes

1 It may be the case that if CMD patients turn out not to be persons (on some agreed upon criteria for personhood), we owe them less than we would persons. For example, if forced to decide between alleviating the suffering of a CMD patient, or a person, we ought to choose the person, insofar as their personhood provides a stronger reason to prevent their suffering than the sentient interests of the CMD patient. Nevertheless, I argue that even if CMD patients aren’t persons (and I leave open that question here), their sentient interests still matter morally, and we would need sufficiently strong moral reasons opposing the alleviation of their suffering to justify failing to do so. In the absence of such reasons, we ought to alleviate their suffering.

2 For a related discussion of precautionary reasoning with respect to personhood, see [18].
